# The Price of Play: Self-Organized Infant Mortality Cycles in Chimpanzees

**DOI:** 10.1371/journal.pone.0002440

**Published:** 2008-06-18

**Authors:** Hjalmar S. Kuehl, Caroline Elzner, Yasmin Moebius, Christophe Boesch, Peter D. Walsh

**Affiliations:** Max Planck Institute for Evolutionary Anthropology, Leipzig, Germany; Centre for DNA Fingerprinting and Diagnostics, India

## Abstract

Chimpanzees have been used extensively as a model system for laboratory research on infectious diseases. Ironically, we know next to nothing about disease dynamics in wild chimpanzee populations. Here, we analyze long-term demographic and behavioral data from two habituated chimpanzee communities in Taï National Park, Côte d'Ivoire, where previous work has shown respiratory pathogens to be an important source of infant mortality. In this paper we trace the effect of social connectivity on infant mortality dynamics. We focus on social play which, as the primary context of contact between young chimpanzees, may serve as a key venue for pathogen transmission. Infant abundance and mortality rates at Taï cycled regularly and in a way that was not well explained in terms of environmental forcing. Rather, infant mortality cycles appeared to self-organize in response to the ontogeny of social play. Each cycle started when the death of multiple infants in an outbreak synchronized the reproductive cycles of their mothers. A pulse of births predictably arrived about twelve months later, with social connectivity increasing over the following two years as the large birth cohort approached the peak of social play. The high social connectivity at this play peak then appeared to facilitate further outbreaks. Our results provide the first evidence that social play has a strong role in determining chimpanzee disease transmission risk and the first record of chimpanzee disease cycles similar to those seen in human children. They also lend more support to the view that infectious diseases are a major threat to the survival of remaining chimpanzee populations.

## Introduction

Human childhood diseases are renowned for their tendency to exhibit annual and supra-annual cycles [1]–[3]. In attempting to explain this cycling researchers historically looked to environmental influences on immune function. Although environmental affects on immunocompetence and disease mortality rate are widespread and well established [4], [5], what has recently come into focus is the extent to which the dynamics of epidemic disease are also driven by the influence that social connectivity has on disease transmission rates [1], [2], [6], [7].

Some of the most pervasive effects of social connectivity on pathogen transmission involve childhood diseases. In the course of activities such as social play, young children tend to come more often into close physical contact than do adults [8]. In addition, during play young children engage in high risk behavior such as oral contact with other children and/or fomites (e.g. toys). Attendance at schools and day care centers also brings children into close proximity to more potential sources of infection than is typical for adults. Consequently, attendance at schools and daycare centers is a strong predictor of per capita disease risk amongst young children [9]–[11]. This is particularly true for respiratory diseases, which are easily spread through casual contact [10]. What's more, the seasonal fluctuations in social connectivity produced by school holidays have been implicated as drivers of the seasonal and supra-annual cycling of childhood respiratory diseases [7], [11], [12], which, in developing countries, are the leading cause of mortality amongst children under five years of age [13], [14].

**Figure 1:**
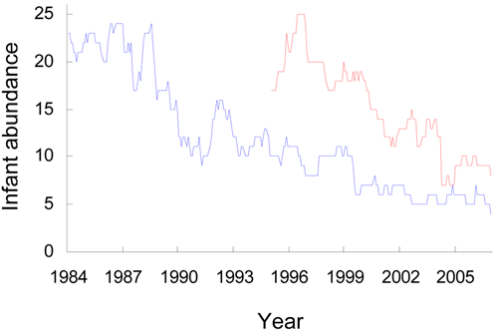
Time series of infant abundance. Number of 0–5 year old infants in North Group (blue) and South Group (red).

Although the consequences of social connectivity for disease dynamics are now increasingly well understood in humans, observations on disease dynamics in our closest relatives, chimpanzees, are very limited. Therefore, we have little sense of the extent to which the social connectivity effects that are so important to the epidemiology of human childhood disease dynamics also drive the dynamics of disease in chimpanzees. Here, we use long term behavioral and demographic data from two chimpanzee communities at our study site in Taï National Park, Côte d'Ivoire [15] to examine whether the elevated social connectivity of chimpanzee infants and, particularly, their high play rates result in disease dynamics similar to those seen in human children [1], [11], [12]. We have previously shown that two human respiratory viruses have, in recent years, caused repeated outbreaks with very high morbidity and substantial infant mortality [16], [ see also 17]–[19]. We do not have similar etiological data for the many earlier years of the study. However, several aspects of the demography of the two communities suggest that a long history of respiratory disease impact has contributed prominently to substantial declines in the size of the two communities [16] (Fig. 1, see also discussion).

In our present analyses we use demographic data to infer whether social connectivity is an important driver of infant mortality dynamics. In particular, we examine to what extent infant per capita mortality rates and changes in infant abundance are predicted by rates of play. We also look for evidence of periodicity in infant abundance and mortality rate. Periodic cycling is a classic signature of respiratory disease outbreak dynamics in human children [1]–[3], [11], [12]. Finally, we examine whether seasonal variations in mortality rate are better explained in terms of seasonal fluctuations in play rates or seasonal variation in external factors such as food availability and rainfall. Although our limited sample sizes prohibit overly strong conclusions, many complementary results point to infant play as a strong determinant of disease transmission and infant mortality dynamics in Taï chimpanzees.

## Materials and Methods

### Study System

Our data come from two habituated chimpanzee communities under continuous and ongoing observation in Taï National Park, Côte d'Ivoire. Habituation of the “North Group” started in 1979 whereas habituation of the second community, the “South Group”, began in 1989. Here we use data only from years in which each community was already well-habituated: North Group 1984–2006; South Group 1995–2006. Both groups were monitored on most days during the study period, although only a minority of individuals was observed on any given day. We recorded the identity of all individuals observed on each day, immigration and emigration of females, births, and deaths (for details see [15]). Because the exact date of each demographic event was not always known, we pooled observations into monthly bins and assigned each event to the last month in which the individual was observed alive (deaths and emigrations) or the first month in which the individual was detected (births and immigrations). In cases in which there was a gap in observations, we assigned the event to the midpoint date of the gap. We used daily focal follows of individuals to collect behavioral data, i.e. time spent playing by infants. Age classes of infants (0–5 years), and juveniles (5–10 years) were defined following ref. [15].

### Habituation effect

We evaluated whether infant and juvenile survival rate increased in the years after habituation with a GLM analysis. In the baseline model, annual survival rate of each individual below the age of 120 month was assumed to be constant across all years of the study. We compared the baseline model to a model in which annual survival rate differed between periods (first six years after habituation vs. rest of time series). We then tested for a density dependent effect by including community size as a covariate. Finally, we estimated a model including both period and community size effects. Relative support for models was evaluated in terms of Akaike's information criterion (AIC) [20].

### Infant Play Behavior and Mortality Rates

We derived age specific play rates for each monthly age class (1–60 months) by calculating the average proportion of one minute observation bouts during which infants played. We estimated age-specific mortality as the number of infant deaths in a given monthly age class (1–72 month) divided by the total number of infants reaching that class. We made this estimate separately for North and South Group. We then used the Pearson Product Moment Correlation to compare age specific mortality patterns in the two communities.

### Autocorrelation and crosscorrelation analysis

We used autocorrelation/ crosscorrelation analyses to test for periodic behavior in the demographic time series data (birth, death, abundance). To reduce stochasticity (e.g. due to death date uncertainty) we pooled data into four month bins. Because the communities declined in size over time, we log transformed the binned data and calculated the rate of change in abundance (log(N_t_)−log(N_t−1_)) in order to get a stationary time series [21]. We then used the autocorrelation function in SPSS version 13 (SPSS Inc. Chicago) to estimate autocorrelation coefficients, and for the death vs. birth time series the crosscorrelation function.

### Covariate model predicting infant mortality

To evaluate the influence of demographic, social and environmental factors on chimpanzee infant mortality, we developed an age structured generalized linear model with the number of deaths per unit time as the dependent variable. As demographic predictors of mortality rate we included both a density dependent effect (infant abundance) and two density independent effects (neonatal and postweaning mortality [22]). In order to include the weaning phase we extended this analysis to the age of 78 months. As a measure of infant social connectivity, we used an index of community-wide playfulness (the product of infant abundance in each monthly age class and age-specific playfulness, summed across age classes). As environmental covariates we used monthly mean rainfall at the study site, an index of the El Niño Southern Oscillation ([23], downloadable at http://www.cdc.noaa.gov/people/klaus.wolter/MEI/), and an index of fruit abundance [24, and N'Guessan et al. unpublished data]. To represent a neonatal mortality peak, we added a term treating mortality rate as inversely proportional to age. To represent a weaning mortality peak we added a term treating mortality rate as inversely proportional to the absolute value of an infants difference in age from the mean interbirth interval (male = 71.4 months, female = 67.1 months, [15]).

We assumed that the probability of infant death in a given month was a logistic function of the predictor variables, so that survival probability (*p_m_*) for month *m* was
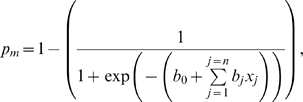
where *b_0_* is a constant and *b_j_* is the coefficient for predictor variable *j*. In order to reduce the effect of uncertainty in death date, we compared model predictions to the number of deaths observed in four month bins, rather than in single months. Thus, the number of infants of age *i* alive at the beginning of a bin starting in month *t* but dead four months later (*D_ti_*) was just
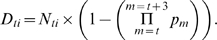
Summing across all initial infant ages (*i* = 1..78) gives the total number of infant deaths predicted for the four month period
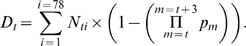
We estimated parameter values using maximum likelihood methods and ranked models using AIC (Akaike's information criterion) [20]. Model fitting was done in R [25] using the ‘optim’ function (log link and negative binomial error function).

## Results

### Habituation effect

**Figure 2:**
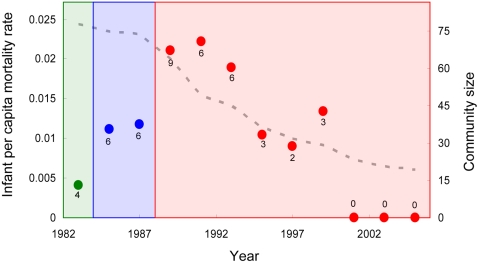
Infant per capita mortality rate for North Group (dots). Each data point represents the monthly per capita mortality rate for infants averaged over a two year interval. Numbers below each dot are numbers of infant deaths. Each colored block represents a distinct period of human presence and proximity. First block (green) is early habituation (before 1984) with only two researchers present. Per capita mortality rate is very low. In second period (blue) chimpanzees are habituated well enough to be followed and only two researchers are present. Per capita mortality rate increases. In 1988 field assistants and additional researchers join study. Per capita mortality rate increases further, then gradually decreases as community size (dashed gray line) decreases, suggesting strong density dependence of infant mortality rate.

If the increasing proximity between humans and chimpanzee resulted in increasing rates of human disease spillover, one would expect to see an effect of the degree of habituation on survival rate. GLM analysis confirmed this expectation with survival rate lower in the years after habituation (Table 1). The effect was strongest for infants, whose per capita mortality risk increased fivefold (Fig. 2).

**Table 1:** GLM results on infant and juvenile survival rates.

model	const	period	size	AIC
C	1.86	-	-	588.9
C+P	2.35	−0.63	-	585.0
C+N	5.77	-	−5.26	549.0
C+P+N	9.92	−1.99	−8.78	508.8

C = baseline model with constant annual survival rate of each individual less than 120 months old. C+P = survival rate for first six years different than for remainder of study period. C+N = survival rate proportional to community size. C+P+N = both period and community size effects.

### Seasonality

**Figure 3:**
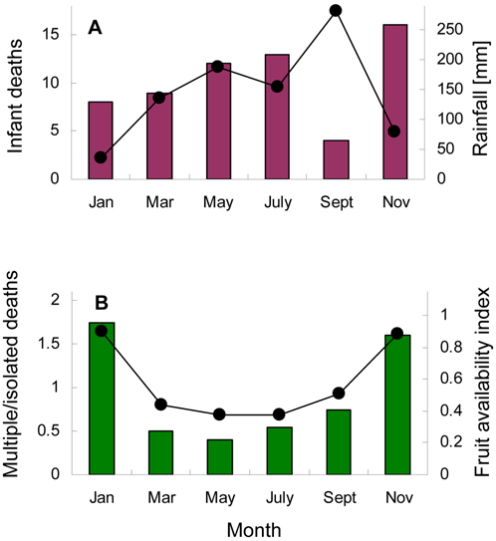
Seasonality of infant death rate. A) Rainfall (line) seasonality was not a good predictor of the number of infant deaths (red bars; same month: Pearson Correlation, R^2^ = 0.233, p = 0.332, n = 6). Bimonthly averages are from rain gauge samples taken at the site between 1987 and 2003. Infant deaths are pooled across North and South Groups. B) Fruit availability (green bars) was a very good predictor of the ratio of the number of deaths in multiple mortality events to the number of isolated deaths (R^2^ = 0.98, p<0.001, n = 6). Fruit availability index calculated from data in ref. 37 and unpublished data provided by A. N'Guessan.

If environmental factors were strong drivers of infant mortality dynamics one might expect to see strong peaks in mortality rate corresponding to either seasonal environmental fluctuations or supra-annual cycles such as those captured by the El Niño Southern Oscillation Index [26]. However, we observed no seasonal clumping of infant mortalities (permutation test, p = 0.45). We also found no significant relationship between infant mortality rate and the average monthly values of either rainfall (Fig. 3a) or an index of the availability of fruit (Pearson correlation, R^2^ = 0.002, n = 12, p = 0.88).

Although the average number of infant deaths per calendar month did not vary with fruit availability, there were indications that the cause of death did. Fruit availability was strongly correlated with the amount of time infants spent playing (Pearson Correlation, R^2^ = 0.5, n = 12, p = 0.01) and the number of different partners each infant played with (Pearson Correlation, R^2^ = 0.62, n = 12, p = 0.002). Both measures of play rate more than doubled during peak fruiting season (October–February). The possibility that high social connectivity might facilitate infectious disease outbreaks led us to predict that multiple mortality events should be more likely in the peak fruiting season while isolated deaths from food stress should be more likely in months of low fruit availability. As predicted the ratio of multiple to isolated mortality events was positively correlated with fruit availability (Fig. 3b).

**Figure 4:**
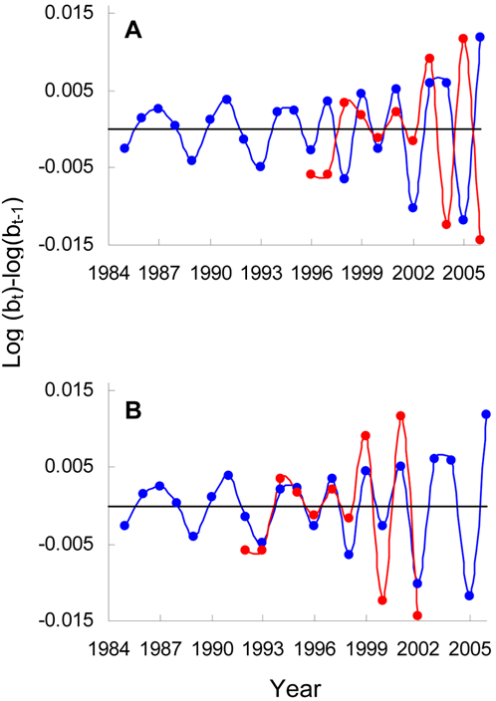
Time series of birth rates. A) Rates of change in birth rate are plotted in terms of the difference between each year (t) and the preceding year (t−1) in the logarithm of the relative number of births (log(b_t_)−log(b_t−1_)). Time series for North Group (blue) and South Group (red) were not correlated (R^2^ = 0.132, n = 12, p = 0.14). B) If the South Group time series is shifted backwards in time to the point at which its initial community size matches that of North Group, the two time series are strongly correlated (R^2^ = 0.7, n = 12, p = 0.0006) and stay in phase for all 11 years.

Finally, an indirect test for supra-annual environmental forcing involves the synchrony of the two communities. If broad scale environmental factors were driving chimpanzee mortality dynamics, then one might expect the two neighboring communities to show correlated fluctuations in infant abundance. We found no such correlation (Fig. 4a). In fact, fluctuations in the infant abundance time series were much better explained in terms of intrinsic (density dependent) dynamics than in terms of extrinsic forcing. This can be seen by shifting the time series for the later habituated South community back in time until its initial abundance matches the abundance of the North Group (Fig. 4b). The time series are not only correlated, they remain in perfect phase for 12 years.

### Infant Play Behavior and Mortality Rates

**Figure 5:**
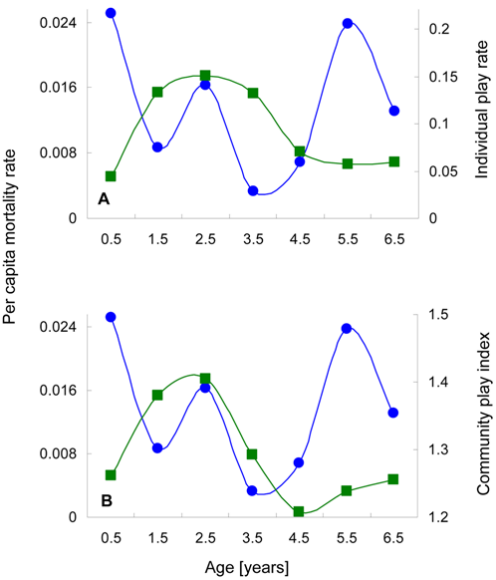
Play and age specific mortality rate. A) Per capita infant mortality rate (blue) shows a strong peak at 2.5 years, the same age as a peak in the proportion of each day that infants spend in social play (green). Mortality rates estimated using data from both communities. Additional mortality rate peaks occur during the neonatal period and around the time of weaning (5–6 years). Variation in the number of play partners shows the same shape (e.g. the average number of play partners increased from less than three during the first year of life to almost seven at 2.5 years; data from North Group, 1992–1994). B) An index combining the abundance and playfulness of infants (green) has the same peak as the individual play rate but drops more sharply because outbreaks that occur when large cohorts of infants reach 2–3 years of age predictably reduce infant abundance (and therefore the value of the play index).

The alternative to extrinsic, environmental forcing of infant mortality dynamics is that the dynamics are self-organized. That is, some intrinsic property of individual chimpanzee ontogeny or behavior reliably pushes community-wide mortality patterns into a predictable trajectory: a dynamical attractor. In the context of disease transmission, the factors that structure rates of social contact are an obvious place to look for mechanisms of self-organization. The ontogeny of social play seems particularly likely to be important because play is the most common venue for direct physical contact or proximity close enough to create a high risk of aerosol or fomite transmission. Both the amount of time infant chimpanzees spend in social play and the number of different partners they play with rise sharply for their first two years, reaching a peak at about 2.5 years of age (Fig. 5a). Play rates then gradually subside. Peak play infants spend more than twice as much time playing and play with more than twice as many partners as infants either two years younger or two years older. Peak play infants also spend twice as much time in social play as adults spend in close contact activities such as grooming [15], [27]. Relative respiratory disease transmission risk for infants is further elevated by the fact that they frequently engage in sham biting and oral manipulation of objects [28].

The peak in infant play is intriguing because it is centered almost exactly on a peak in infant per capita mortality rate. The peak at 2.5 years is joined by two other major peaks in per capita mortality rate, a neonatal peak and a peak at about six years. There is no obvious life history event that corresponds to the mortality peak at 2.5 years, other than the peak in play. In contrast, the two other peaks correspond to clear life history watersheds. Neonatal mortality peaks are common in primates and may be caused by birth complications, congenital defects, poor mothering, or the weak immunity of newborns [15], [29]. The peak at 5–6 years occurs around weaning so it might plausibly be attributed to food stress. Just as plausible is the possibility that the cessation of the maternal antibody subsidy provided by breast milk makes freshly weaned juveniles particularly susceptible to infectious disease. Maternal antibodies in breast milk suppress antibody production in human children and their termination at weaning results in a spike in respiratory disease infection [22], [30].

### Play and Cohort Cycling

**Figure 6:**
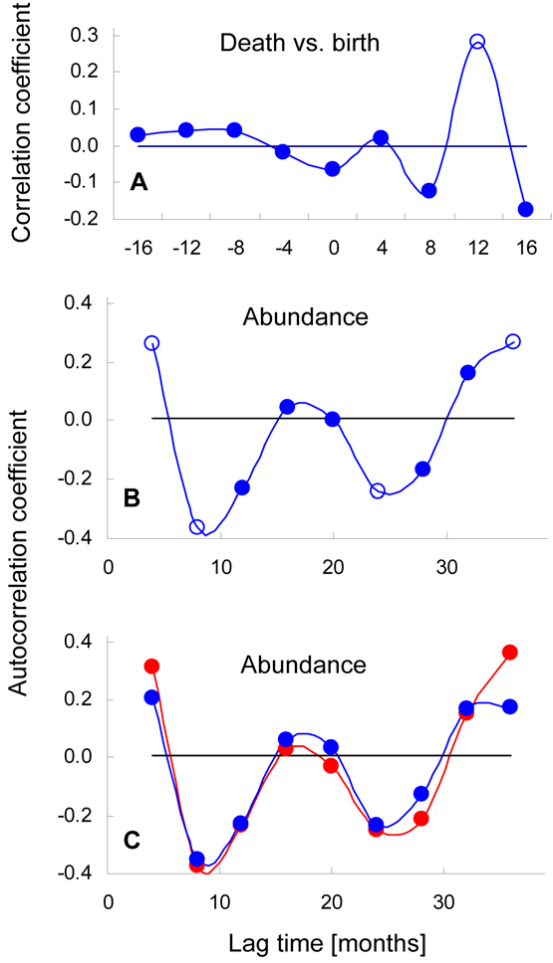
Correlations between infant death rate, birth rate and abundance. A) Infant birth rates are positively correlated with infant death rates at a time lag of about 12 month. B) Abundance of infants age 0–5 years shows significant negative autocorrelation at time lags of 9–12 and 24–27 months, and significant positive autocorrelation at a time lag of 37–40 months (open squares indicate Pearson Correlation p<0.05). Data are pooled across North and South Groups and data points are plotted in four month bins. C) Lag autocorrelation curves for North Group (blue) and South Group (red) were strongly correlated (Pearson correlation, R^2^ = 0.96, n = 11, p<0.0001).

One consequence of the death of multiple infants in a disease outbreak is that the reproductive cycles of their mothers are synchronized. Female chimpanzees typically come into estrous within a few weeks and conceive within a few months after losing an infant, and have a gestation time of about eight months [15]. Consequently, the synchronizing effect of disease outbreaks results in a strong correlation between the number of infant deaths and the number of births 9–12 months later (Fig. 6a).

Now, if the propensity towards respiratory outbreaks in each community was strongly determined by the number of immunologically weak individuals, then one might expect outbreak probability to fall after an outbreak and rise immediately with the introduction of a synchronized cohort of neonates, which have notoriously weak immune systems [29]. Assuming fairly persistent exposure to respiratory pathogens, a new outbreak would follow relatively soon thereafter. In other words, infant mortality rates would cycle on a period of roughly one year.

Infant per capita mortality rates did show the expected neonatal peak (Fig. 5). However, autocorrelation analyses of mortality rate did not show the roughly one year cycle predicted by simple density dependence. Rather, mortality rates showed a strong positive autocorrelation only at 28 months (Pearson correlation, R^2^ = 0.295, n = 65, p = 0.017). Interestingly, the time separation between the neonatal peak in per capita mortality rate and the mortality peak at maximum play age was also 28 months (Fig. 5). What appears to be happening is that the birth of a large, post-outbreak cohort resulted in a small pulse in neonatal deaths. The number of deaths then stayed low for the following two years as infant abundance swelled with the addition of smaller following cohorts. Finally, when the large, post-outbreak cohort approached its peak of social play at 2–3 years, outbreaks became more likely and infant mortality rates again peaked. The cycle then started anew.

**Figure 7:**
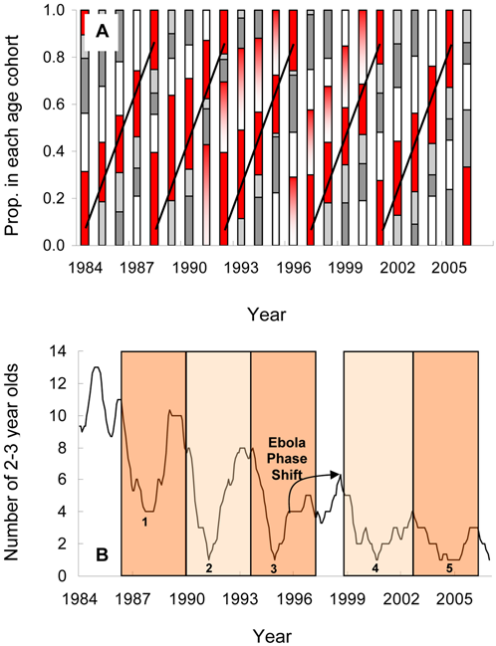
Birth cohort cycling in North Group. A) Proportion of infants in each annual birth cohort through time. Large birth cohorts (red) are separated by 3–4 years. In cases where large birth cohorts occurred in consecutive years (1991 and 1996) the first cohort is shaded red. B) Time series for the abundance of peak play (2–3 year old) infants shows a consistent cycle period of about 3.5 years. A shift in phase resulted from an Ebola outbreak in 1994.

The cyclic nature of cohort structure can also be seen in the time series for the North Group, with large cohorts moving through the community on a regular cycle (Fig. 7a). The regularity of this cycle is even more apparent when the data are plotted in terms of the abundance of only the playful 2–3 year old cohort (Fig. 7b). The period from 1986 to 2006 contains five cycles with clear peaks and troughs in the number of 2–3 year olds. The cycles stay in phase through the 21 year period except for a three year phase shift caused by an Ebola virus outbreak in 1994, which killed all five females who were expected to give birth in 1995–6 (three pre-weaning mothers and two nulliparous immigrants).

**Figure 8:**
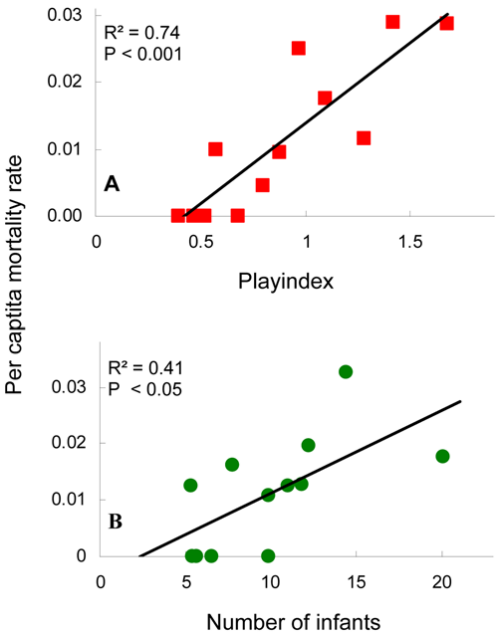
Infant per capita mortality risk as a function of play index (A) and infant abundance (B). Per capita infant mortality rate, infant abundance, and the community-wide play index were calculated separately for each month in the two time series. Data from the two communities were then pooled, sorted by play index or infant abundance, and divided into 12 equal size bins. The 20 monthly estimates within each bin were then averaged. The averaged per capita infant mortality rates were more strongly correlated with values of the play index (R^2^ = 0.75, p = 0.0003, n = 12) than with infant abundance (R^2^ = 0.41, p = 0.025, n = 12).

A critical element in maintaining the strong cohort structure seen in Fig. 7 was likely that respiratory outbreaks at Taï showed a very high morbidity [16]. Even though outbreaks may have been precipitated by the presence of large cohorts of playful 2–3 year olds, mortality during outbreak periods was spread across infant ages. Consequently, the abundance of all infants, not just their distribution amongst cohorts, also showed strong cycling (Fig. 6b). Autocorrelations on infant abundance show this effect clearly. There were strong negative correlations corresponding to the gestation length (pre-outbreak peak to pre-birth trough, post-outbreak trough to post-birth peak) and the interval from birth to peak play age (pre-birth trough to pre-outbreak peak, post-birth peak to post-outbreak trough). And there was a positive autocorrelation in infant abundance (∼37–40 months) at a lag equal to the sum of the two negative lags: i.e. roughly the interval from pre-outbreak peak to pre-outbreak peak or post-outbreak trough to post-outbreak trough. The fact that the two communities showed very similar autocorrelation profiles (Fig. 6c) is highly suggestive of a common mechanism of self-organization, particularly if one remembers that rates of change in births in the two communities were uncorrelated in real time (Fig. 4a). That the period of the cohort cycles (Fig. 7b; ∼3.5 years) is slightly longer than the 37–40 month autocorrelation peak in Fig. 6b,c reflects the tendency of 2–3 year old abundance to “pause” at high levels for six months or more before crashing. That the observed cycling was driven by infant playfulness, rather than just infant abundance, is suggested by the observation that the index of community-wide playfulness was a much better predictor of infant per capita mortality rate than was simple infant abundance (Fig. 8).

The strong cohort structure also generated a closer correspondence between play and mortality risk than might be inferred from the broad peak in individual play rates (Fig. 5). The lag between the rise in play rates and the rise in mortality rates may simply reflect a stochastic “waiting time” before disease introduction into each community. And the slow drop in individual play rates does not convey the tendency for outbreaks that occurred when large cohorts reached peak play age to kill many infants. Thus, even though high individual play rates continued beyond the 2–3 year age class, the community-wide play index followed a sharp downward trajectory very similar to that of mortality rates (Fig. 5b). This result reinforces the idea that the 2–3 year old peak in mortality rate was not just an intrinsic property of individuals but, rather, an emergent outcome of social interactions amongst individuals.

### Combining the Effects

To evaluate the relative strength of extrinsic and intrinsic effects on infant mortality rates we compared a series of generalized linear models that included social, demographic, and environmental variables as predictors. The globally best model was 11.5 AIC units better than a null model assuming constant mortality rate. An index combining infant abundance with infant playfulness was the best covariate predictor of infant mortality rate (Table 2) and was positively correlated with mortality rate. It had the highest cumulative Akaike weight (0.98) and in univariate analyses produced the greatest improvement over a null model assuming constant mortality rate (7 AIC units). Fruit availability was also a good predictor, with high infant mortality rate increasing at times of low food availability. The fruit index produced only 4 AIC units of model improvement when evaluated in a univariate analysis but when combined with play received an Akaike weight of 0.93.

**Table 2:** GLM results for covariate model predicting infant mortality rate.

model	# par	const	play	fai	mei	neo	wean	AIC
play+fai+mei	5	−3.17	1.26	−4.89	−0.20	-	-	126.73
play+fai	4	−0.81	1.23	−10.92	-	-	-	126.78
play+fai+mei+neonatal+weaning	7	0.34	1.39	−15.16	−0.25	2.58	0.36	130.03
play	3	−5.75	1.37	-	-	-	-	131.39
fai	3	0.96	-	−11.80	-	-	-	134.24
null	2	−4.19	-	-	-	-	-	138.30

Model = variables in the model; #par = number of parameters (includes also shape parameter, which is not shown here), the coefficients for the constant (const), and the variables (playindex (play), fruit availability index (fai), Multivariate El Niño Southern Oscillation index (mei), neonatal mortality (neo), weaning (wean)), and AIC. Only major effects shown. For full results see Supporting Materials (Table S1).

There was only weak support for climate forcing, with the El Niño Index appearing in models with a combined Akaike weight of only 0.47. Despite strong neonatal and post-weaning peaks in the analysis of per capita mortality rate (Fig. 4), neither the number of neonates nor the number of post-weaning juveniles was a good predictor of the number of deaths in a given period (cumulative Akaike weights 0.29 and 0.33, respectively). The lack of stronger effects may reflect the high morbidity of respiratory disease outbreaks at Taï. Although high 2–3 year old abundance may have been a trigger for outbreaks, individuals appeared to die in proportion to their (age-specific) immune susceptibility. Because a large proportion of deaths (roughly half) occurred during outbreaks, the number of highly susceptible neonates and post-weaning juveniles dying at a given time was better predicted by the frequency of peak play 2–3 year olds than it was by their own frequency. This effect may have been magnified for the post-weaning juveniles in that the members of a large cohort that survived one outbreak cycle tended to reach the post weaning year just as the following large cohort reached peak play age.

## Discussion

Perhaps the most surprising result of our analyses was the great regularity of cycles in infant mortality and abundance. This regularity was surprising to us because of the stochasticity inherent in the small sample sizes involved in our study. That cycles were so regular despite this stochasticity suggests a very strong structuring mechanism.

Our observations suggest that this structure was not extrinsically imposed but self-organized as a consequence of three relatively invariant aspects of chimp demography. First, outbreaks that killed multiple infants synchronized the reproductive cycles of their mothers. Second, after a female lost a baby, there was very low variance in the time to estrous and conception as well as in the gestation period [15]. Third, infant play rates followed a predictable rise to a peak at age 2–2.5 years. Consequently, the death of a large group of infants in one outbreak reliably resulted in a large cohort of highly playful, socially connected infants about three years later: in other words, ideal conditions for propagation of a new outbreak. In this context, it must again be emphasized that these peak play infants spend twice as much time playing and play with twice as many partners as other subadults and play twice as much as adults engage in close contact activities such as grooming. Peak play infants are ideal super spreaders [10].

The major caveat to our conclusions is the fact that definitive etiological data on respiratory pathogen identity were only available in the later years of the study. This prevents us from arguing conclusively that respiratory disease was the primary driver of mortality in the earlier period. We are persuaded that respiratory disease was the major driver, in part, because all of the other plausible sources of infant mortality fail to explain one or more aspects of our observations. For example, although climate cycling might plausibly affect either immune function (through its affects on thermal stress [31], food stress [32], [33], or mucosal membrane function [34], [35]), rates of pathogen spillover from other hosts [36], or duration of pathogens persistence in the environment [37], we found little evidence of such effects. Not only did climate variables do a poor job of predicting supra-annual mortality cycles, infant population cycling in the two chimpanzee communities were not even in phase. Respiratory pathogens such as respiratory syncytial virus also typically cycle through human populations on a shorter period (1–2 years) than the cycles seen in our study chimpanzees [38]. Thus, spillover from cyclic human respiratory epidemics does not seem a likely driver.

The strong dependence of per capita mortality rates on infant density was also consistent with epidemic respiratory disease but not with vector borne disease because chimpanzee communities are too small to maintain persistent circulation of diseases such as malaria. Furthermore, the study chimpanzees do not live near potential sources of water borne disease (i.e. villages) and we never saw a high prevalence of waterborne disease symptoms (e.g. diarrhea). And, although Ebola [39] and Anthrax [40] have been isolated in Tai, long term screening of the chimpanzee population did not lead to the repeated identification of anything other than respiratory pathogens, which were isolated from four different outbreaks during the last nine years [16].

Non-disease sources of mortality such as starvation or predation also show inconsistencies with the observed patterns of infant mortality. For instance, although fruit scarcity was positively associated with infant mortality rate, this seasonal peak involved isolated deaths. In contrast, multiple mortality events were actually most common in the season of high fruit availability. And, as mentioned above, there was little support for an association between extreme climate fluctuations (e.g. El Niño) and peaks in mortality rate, as one might expect if starvation were a major force structuring infant mortality cycles.

There are also good reasons to doubt that predation was a major driver of the observed infant mortality cycles. Cyclic predator-prey dynamics can arise if a predator species is largely dependent on a single prey species [41]. However, the primary chimpanzee predator, leopards, feed on many different prey species, each with a different demography [15], [42]. Besides, leopards mature so slowly that it would be impossible for major fluctuations in adult leopard density to occur on the short (∼three year) time scale on which chimpanzee infant abundance showed major fluctuations. This leaves behavioral mechanisms as the only plausible explanations of how predation might drive infant mortality cycles. For example, if leopards switched to hunting infant chimpanzees only when they achieved high densities, then predation could in principle drive cycles in infant abundance. However, leopard prey switching would have to be remarkably abrupt to explain both the cyclic pattern of mortality and the large size of many of the observed mortality events (e.g. four deaths in a single month in 1988). One might also expect that the increasing human presence during the early part of the study would have discouraged leopard predation rather than resulting in higher infant mortality rates (Fig. 2). And one would also have to assume that the driving effect of predation stopped abruptly in 1999, as all multiple mortality events recorded thereafter showed high prevalence of respiratory symptoms and supporting diagnostic samples [16].

In sharp contrast, many different aspects of infant mortality patterns at Taï are consistent with respiratory disease: including many aspects other than infant mortality cycling. For example, respiratory symptoms were frequently observed at high prevalence in the era before pathogen sampling was initiated [16]. The observations that mortality rate was density dependent and that multiple mortality events peaked in the season of high social connectivity are also consistent with infectious disease outbreak. In addition, the demographic profile for the multiple mortality events of unconfirmed origin was remarkably similar to that of confirmed respiratory disease outbreaks, which differed from confirmed outbreaks of other diseases (i.e. Ebola, and Anthrax [16]). The observed post weaning peak in juvenile mortality is consistent with loss of the protection provided by maternal antibodies in breast milk: a phenomenon that has been documented for many respiratory diseases including the viruses that caused several confirmed respiratory outbreaks in Taï chimpanzees [30]. The fact that infant mortalities rose sharply in each community when better habituation allowed closer approach by more researchers is consistent with pathogen spillover from researchers (Fig. 2), as is the global provenance of the pathogens detected during outbreaks [16]. Individually these observations each have caveats and counter-arguments. However, we believe that as an ensemble they make a convincing case that respiratory disease was the primary driver of infant mortality dynamics at Taï.

Our results show for the first time that social connectivity plays the same central role in structuring disease cycling in young chimpanzees that it does in human children. However, the mechanisms of cycling in Taï chimpanzees were different than those typically observed in modern humans. In humans, seasonal patterns of school attendance generate seasonal fluctuations in disease exposure and, therefore, specific immunity that modulate outbreak probability [2], [12]. Specific immunity undoubtedly played a role in disease cycling at Taï, particularly in determining the disease susceptibility of neonates and post-weaning juveniles. However, high infant mortality rates during outbreaks produced strong cycles in infant abundance, not just in the prevalence of specific immunity. And it was the number of infants, rather than just their immune status, that appeared to drive outbreak cycling. Furthermore, although seasonality in social connectivity did appear to affect outbreak probability in a way analogous to humans, the age-specific playfulness of infant chimpanzees appeared to have an even stronger effect. The role of specific immunity might be more rigorously probed using a dynamical modeling approach rather than the static statistical approach taken here. Unfortunately, the relatively short length of the data time series and the small sizes of the chimpanzee communities provide little statistical power for implementing such an approach.

As for the implications of our results for future research, we are hoping that our sample does not grow much larger. Once we recognized that respiratory disease transmission might be a problem, we instituted stringent hygiene precautions such as the wearing of masks, and prohibition of individuals with respiratory symptoms from entering the forest [16]. Only time will tell how effective these measures will be.

**Figure 9:**
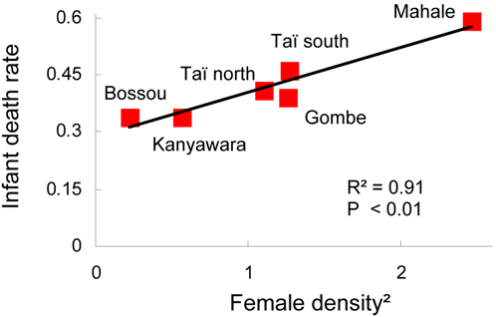
Infant death rate as a function of female density. Included are six communities at five study sites for which infant death rates (survivorship) are published [43] and where females have been followed closely over a long period. Female densities were taken from [19], [44], and serve as proxy for infant density (for which no published data were available).

However, we hope that the result from Taï will be taken seriously elsewhere. Respiratory symptoms have been observed at several other sites where gorillas and chimpanzees are habituated for research or tourism [17]–[19]. We found that the strong density dependence that typified infant mortality dynamics within Tai was also evident in a cross site comparison including all chimpanzee research sites for which we could find published data on infant survivorship (Fig. 9). The proportionality of infant death rate to the square of infant density is particularly suggestive because the probability that a disease spillover event amplifies throughout a population is closely related to the rate of contact between individuals, which in a well mixed population increases with the square of density [1].

A detailed meta-analysis of demographic, behavioral, and environmental data from several field sites with declining and non-declining chimpanzee populations would be a promising next step to evaluate the findings of this study independently. Our guess is that a retrospective analysis of demographic data from the several sites that have experienced serious declines might show a demographic signature of respiratory disease similar to the one observed at Taï.

Such a cross-site comparison would help in the further evaluation of alternative or complementary hypotheses on the role of climate forcing, food stress, or predator pressure on chimpanzee population dynamics. Site specific differences in the history of human-chimpanzee contact might also provide better insight into both the role of research and tourism in determining pathogen spillover rates and the extent to which acquired immunity in chimpanzees influences the outcome of pathogen spillover. This issue may become even more important in the near future, given the increasing fragmentation of chimpanzee habitat and, thus, increasing proximity of human and chimpanzee populations.

Finally, we strongly encourage all sites where apes are habituated to close human contact to take both appropriate precautions [16] and detailed data on ape demography, clinical symptoms, and disease etiology. Whenever possible, post mortem necropsy should be conducted. Field site staff health monitoring might also prevent new disease outbreaks and allow the origin of any outbreaks that do occur to be identified. We hope that this study will lead to a debate about the ethics and consequences of long-term field sites for research and tourism, and about appropriate measures for the prevention of disease transmission.

## Supporting Information

Table S1 This file contains the full GLM results, from which only a selection is shown in the manuscript.
(0.03 MB XLS)
